# An updated version of the Madagascar periwinkle genome

**DOI:** 10.12688/f1000research.129212.1

**Published:** 2022-12-21

**Authors:** Clément Cuello, Emily Amor Stander, Hans J. Jansen, Thomas Dugé De Bernonville, Audrey Oudin, Caroline Birer Williams, Arnaud Lanoue, Nathalie Giglioli Guivarc'h, Nicolas Papon, Ron P. Dirks, Michael Krogh Jensen, Sarah Ellen O'Connor, Sébastien Besseau, Vincent Courdavault

**Affiliations:** 1 EA2106 Biomolécules et Biotechnologies Végétales, Université de Tours, Tours, 37200, France; 2 Future Genomics Technologies, Leiden, 2333BE, The Netherlands; 3 Present address: Centre de Recherche, Limagrain, Chappes, 07745, France; 4 IRF, SFR ICAT, Univ Angers, Univ Brest, Angers, 49000, France; 5 Novo Nordisk Foundation Center for Biosustainability, Technical University of Denmark, Kongens Lyngby, 2800, Denmark; 6 Department of Natural Product Biosynthesis, Max Planck Institute for Chemical Ecology, Jena, 07745, Germany

**Keywords:** Monoterpene indole alkaloids, Catharanthus roseus, Apocynaceae

## Abstract

The Madagascar periwinkle, *Catharanthus roseus*, belongs to the *Apocynaceae* family. This medicinal plant, endemic to Madagascar, produces many important drugs including the monoterpene indole alkaloids (MIA) vincristine and vinblastine used to treat cancer worldwide. Here, we provide a new version of the *C. roseus* genome sequence obtained through the combination of Oxford Nanopore Technologies long-reads and Illumina short-reads. This more contiguous assembly consists of 173 scaffolds with a total length of 581.128 Mb and an N50 of 12.241 Mb. Using publicly available RNAseq data, 21,061 protein coding genes were predicted and functionally annotated. A total of 42.87% of the genome was annotated as transposable elements, most of them being long-terminal repeats. Together with the increasing access to MIA-producing plant genomes, this updated version should ease evolutionary studies leading to a better understanding of MIA biosynthetic pathway evolution.

## Introduction

The Madagascar periwinkle, *Catharanthus roseus* (L.) G. Don, is an *Apocynaceae* plant native to Madagascar. *C. roseus* produces several specialized metabolites including monoterpene indole alkaloids (MIA; O’Connor and Maresh, 2006). These molecules are produced by plants to face biotic and abiotic pressures accounting for their wide range of bioactive properties ( Dugé de Bernonville *et al.*, 2015). Above all, MIAs produced by *C. roseus* are well-known for being part of the human pharmacopoeia against cancer, such as the well-known vinblastine and vincristine, and other MIA derivatives, including vinorelbine ( O’Connor and Maresh, 2006).

Due to its high economic importance, *C. roseus* has extensively been studied within the last three decades becoming the model species for MIA biosynthetic pathway studies (see Pan *et al.*, 2016 and Kulagina *et al.*, 2022 for extensive review). *C. roseus* genome was firstly sequenced in 2015 ( Kellner *et al.*, 2015). Recently, a more contiguous version (v2) was generated to ease inter-species genomic comparison ( Franke *et al.*, 2019). To date, *C. roseus* genome sequencing and assembly did not benefit from the development of third generation sequencing technologies that lead to more contiguous genome ( Jiao and Schneeberger, 2017). Thanks to these new technologies, we present here an even more contiguous genome assembly. This updated version (v2.1) should ease inter-species studies in order to better understand the diversification of MIAs and the evolution of their biosynthetic pathways.

## Methods

### Sample collection, DNA extraction and sequencing

*C. roseus* cv ‘SunStorm ^®^ Apricot’ seeds (variety ID: 70001114, Syngenta flowers, Basel, Switzerland) were greenhouse-grown at the University of Tours for 1 month before sampling. DNA was extracted from *C. roseus* leaves using Qiagen Plant DNeasy kit (ID: 69204, Qiagen, Hilden, Germany) following the manufacturer’s instructions. Illumina sequencing library were constructed using the TruSeq DNA PCR-free kit (ID: 20015962, Illumina, San Diego, USA) and sequenced in paired-end mode (2 × 150 bp) by Eurofins Genomics (Les Ulis, France) using Illumina NextSeq500 technology. Future Genomics Technologies (Leiden, The Netherland) constructed ONT library using ONT 1D ligation sequencing kit (SQK-LSK109, Oxford Nanopore Technologies Ltd, Oxford, United-Kingdom) subsequently sequenced on Nanopore GridION flowcell and Nanopore PromethION flowcell (Oxford Nanopore Technologies Ltd, Oxford, United-Kingdom) with the GuPPy (RRID:SCR_022353) version 3.2.6 high-accuracy basecaller. A total of 114,329,683 paired-end reads were obtained from the Illumina HiSeq sequencing, 908,999 and 2,588,997 from the ONT GridION and ONT PromethION sequencing, respectively.

### *De novo* genome assembly

The *C. roseus* genome was assembled by Future Genomics Technologies (Leiden, The Netherlands). After adapters removal using Porechop (RRID:SCR_016967) ( Wick *et al.*, 2017), ONT reads were first assembled into contig using Flye (RRID:SCR_017016) assembler (v.2.5, Kolmogorov *et al.*, 2019) with the following options: --min-overlap 10000 -i 2. Redundant contigs were removed using Purge_haplotigs (RRID:SCR_017616) (v.1.1.0) followed by two rounds of polishing with Illumina paired-end reads using Pilon (RRID:SCR_014731) (v.1.23, Walker *et al.*, 2014).

### Gene model prediction and gene functional annotation

RNA-seq data were retrieved from the NCBI Sequence Read Archive (SRA) (RRID:SCR_004891) database using the following accession numbers: ERS1229288, ERS1229289, ERS1229290, ERS1229291, ERS1229292, ERS1229293, ERS1229294, ERS1229295, ERS1229296, ERS1907920, ERS2396963, ERS2396964, ERS2396965, ERS2396966, SRR20661631. These data were individually aligned to the *C. roseus* genome using HISAT2 (RRID:SCR_015530) (v.2.2.1, Kim *et al.*, 2019). Transcripts were subsequently assembled using the resulting RNA-seq alignments and StringTie (RRID:SCR_016323) (v.2.1.7, Pertea *et al.*, 2015). These individual transcriptomes were further merged using stringtie-merge to a non-redundant set of transcripts. A combination of similarity search using BLASTX (RRID:SCR_001653) and BLASTP (v.2.6.0-1, Camacho *et al.* 2009) against UniProt (RRID:SCR_002380) database (v.2022-10-12) and hmmscan (v.3.1b2, Finn *et al.*, 2011) against the Pfam (RRID:SCR_004726) database was used to assign putative function to each gene model.

### Assembly completeness assessment

The stat program from BBmap (RRID:SCR_016965) tool (v.38.94, Bushnell, 2014) was used to assess assembly quality. Benchmarking Universal Single-Copy Orthologs ( BUSCO v.5.2.2, Simão *et al.*, 2015) (RRID:SCR_015008) with default settings was used to assess genome and gene models completeness using a plant-specific database of 2,326 single copy orthologs (eudicots_odb10). The agat_sp_statistics perl script from the AGAT package (v.0.8.0, Dainat *et al.*, 2022) was used to get the gene models statistics.

### Transposable elements (TE) prediction and annotation

Identification and annotation of transposable elements was determined using extensive *de novo* TE annotator ( EDTA v.1.9.5, Ou *et al.*, 2019) (RRID:SCR_022063) using the sensitive mode. This pipeline annotates long-terminal repeat (LTR) using LTR_Finder (RRID:SCR_015247) (v. 1.07, Xu and Wang, 2007) and LTRharvest (RRID:SCR_018970) included in GenomeTools (RRID:SCR_016120) (v.1.5.10, Ellinghaus *et al.*, 2008); terminal inverted repeat (TIR) using Generic repeat finder (v.1.0, Shi and Liang, 2019) and TIR-learner (v.2.5, Su *et al.*, 2019); and Helitrons using HelitronScanner (v.1.1, Xiong *et al.*, 2014). TE size thresholds are further used to prevent false discoveries. Hence, TIR shorter than 80 bp as well as LTR and Helitrons shorter than 100 bp are considered as tandem repeats and short sequences. To prevent false LTR discoveries, LTR are further filtered using LTR_retriever (RRID:SCR_017623) (v.2.9.0, Ou and Jiang, 2018). TIR candidates are classified as MITEs if not exceeding 600 bp. TIR and Helitrons are further filtered using EDTA advanced filters (see Ou *et al.*, 2019 for details). The genome is then masked using the obtained TE library. Unmasked part of the genome is then scanned by RepeatModeler (RRID:SCR_015027) (v.2.0.1, default parameters, Flynn *et al.*, 2020) to identify non-LTR retrotransposons and unclassified TE missed by structure-based TE identification tools. Finally, EDTA uses the provided CDS sequences to remove gene-related sequences.

## Results

### Genome assembly

*C. roseus* genome was assembled from ONT long-reads using Flye (v.2.5) resulting in a 651.9 Mb assembly distributed across 788 contigs. This assembly was collapsed using purge_haplotigs into 173 scaffolds reducing length to 585,8 Mb but increasing N50 from 10.3 Mb to 12.3 Mb. Assembly polishing was performed twice using Illumina short-reads with pilon (v. 1.23). *C. roseus* final assembly consisted in 173 scaffolds with a total length of 581.45 Mb. Even though *C. roseus* v.2.1 displayed similar BUSCO scores compared to *C. roseus* v.2 based on *Eudicotyledons* Benchmarking Universal Single-Copy Orthologs (BUSCO), this new version v.2.1 turns out to be much more contiguous with a 12 time less contigs and a six-fold larger N50 ( Table 1) ( Cuello *et al.*, 2022).

**Table 1.** Genome assembly metrics.

Version	Assembly size (Mb)	No. of scaff. ^a^	N50 (Mb)	BUSCO scores (genome mode) C [S; D]; F; M ^b^	Protein coding genes	Ref.
*C. roseus* v.2	541.13	2,090	2.58	97.0 [95.5; 1.5]; 1.3; 1.7	34,363	Franke *et al.*, 2019
*C. roseus* v.2.1	581.45	173	12.2	97.1 [94.2; 2.9]; 1.0; 1.9	21,061	This study

*C. roseus*: *Catharanthus roseus*; BUSCO: Benchmarking Universal Single-Copy Orthologs.

^a^ Number of scaffolds.

^b^ BUSCO scores (genome mode) % Complete [% Complete and single-copy; % Complete and Duplicated]; % Fragmented; % Missing (n = 2,326).

### Gene annotation

**Figure 1.**
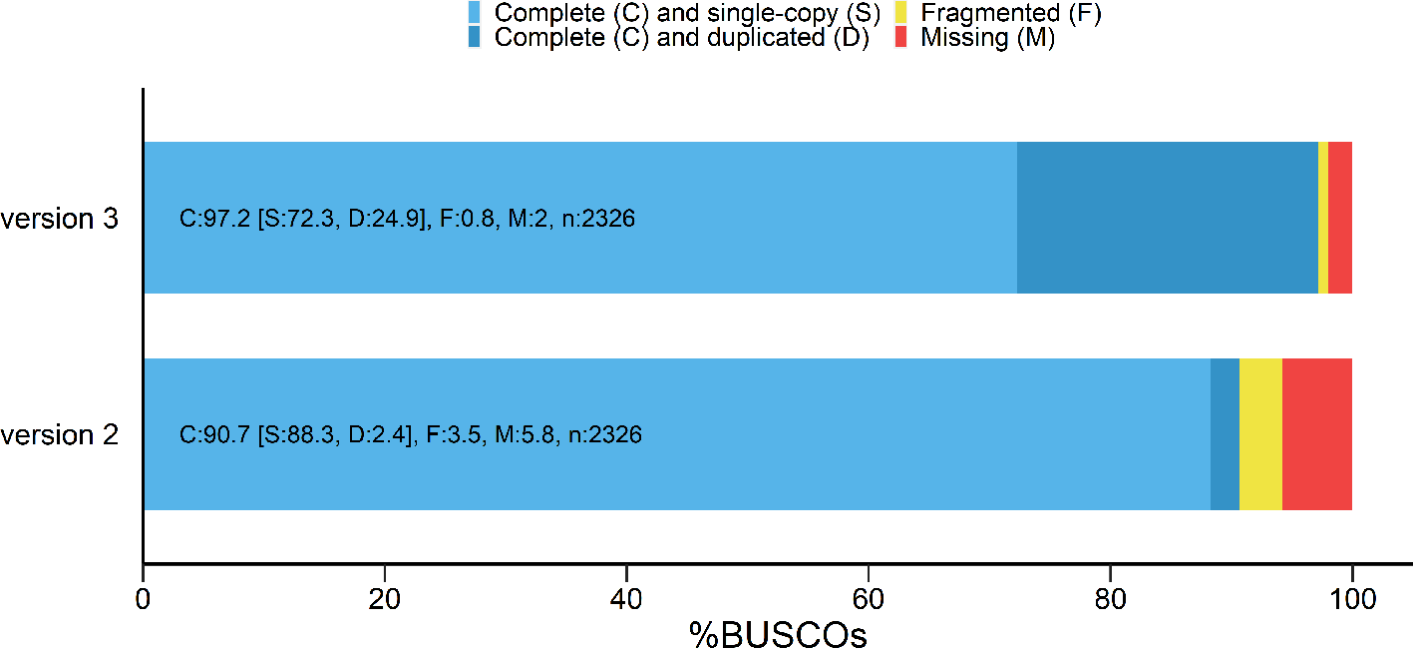
BUSCO scores of the predicted gene set. BUSCO: Benchmarking Universal Single-Copy Orthologs.

RNA-seq based gene model prediction using publicly available data resulted in a total of 21,061 genes. Despite less genes were annotated; a higher BUSCO score was obtained ( Figure 1). The combination of BLASTP and BLASTX against UniProt database and hmmscan against the PFAM database led to the functional annotation of 76.5% of the predicted genes (16,118 of the 21,062 genes, Supplementary Table S1 in *Underlying data* ( Cuello *et al.*, 2022)). All functionally validated MIA biosynthetic genes from *C. roseus* could be found in this new version v.2.1 of the genome with identity and coverage percentage ranging from 95 to 100% and 94 to 100%, respectively, with the exception of *G10H* and *DAT* (Supplementary Table S2-S3 in *Underlying data* ( Cuello *et al.*, 2022)).

### Transposable element annotation

**Figure 2.**
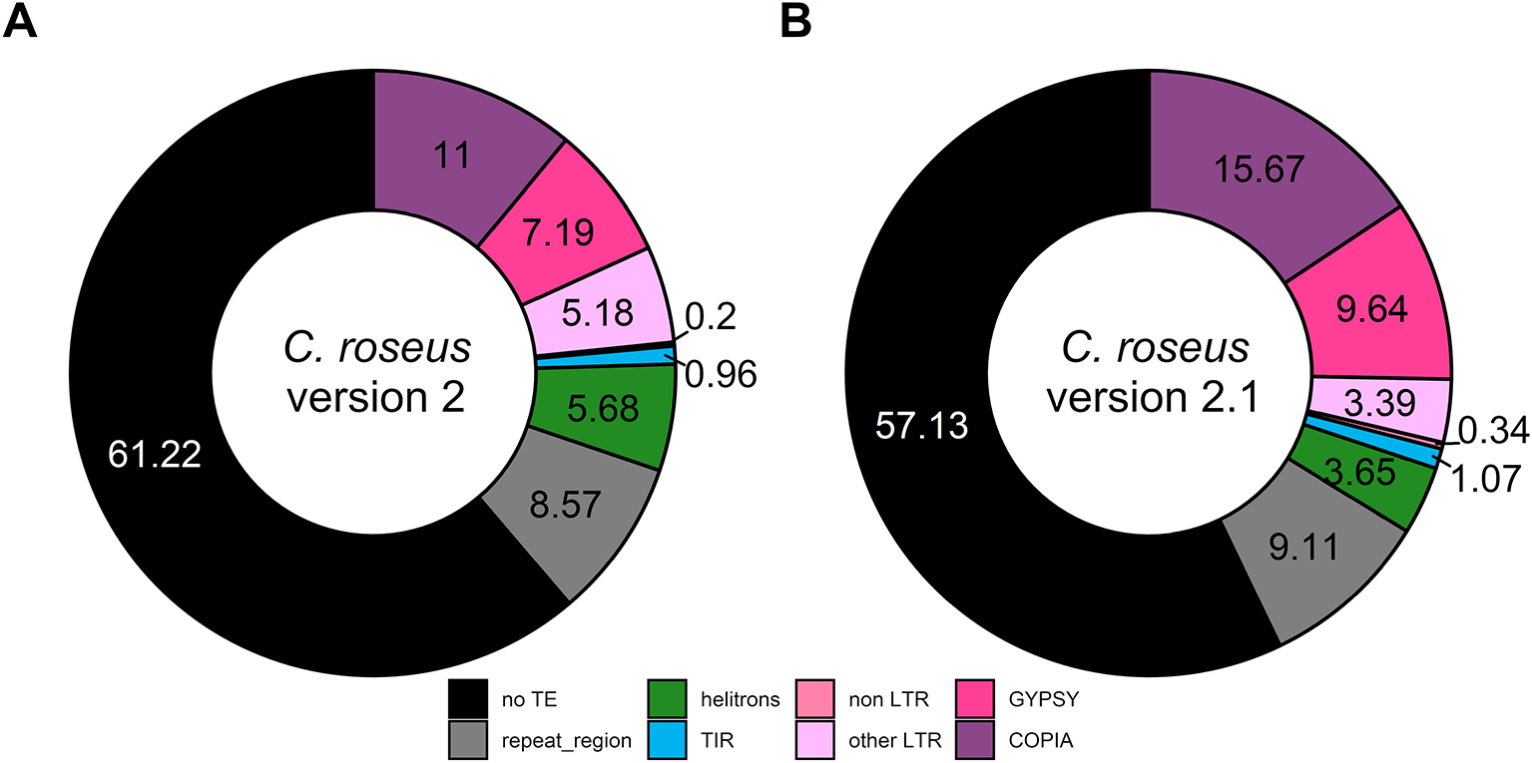
Proportion of transposable element (TE) in *C. roseus* assembly version 2 (A) and version 2.1 (B). TIR: terminal inverted repeat, LTR: long terminal repeat, non LTR: retrotransposons without LTR sequence, other LTR: LTR containing retrotransposons except for *Gypsy* and *Copia.*

Finally, we analyzed TE composition of this updated *C. roseus* genome. While 38.78% of the genome consisted in TE in *C. roseus* v.2, a higher proportion (42.87%) was annotated as TE in this new version (v.2.1) with similar distribution across the different TE families ( Figure 2). It is worth noting that TE proportion of this v.2.1 is closer to the one in its recently sequenced closely related species *Vinca minor* ( Stander *et al.*, 2022).

## Data Availability

BioProject: *Catharanthus roseus* genome sequencing. Raw sequence reads, complete genome. Accession number PRJNA907167, https://identifiers.org/NCBI/bioproject:PRJNA907167 ( Tours University, 2022a). BioSample: Plant sample from *Catharanthus roseus*, Accession number SAMN31953452, https://identifiers.org/NCBI/biosample:SAMN31953452 ( Tours University, 2022b). Figshare: An updated version of *Catharanthus roseus* genome. 10.6084/m9.figshare.21641111 ( Cuello *et al.*, 2022). This project contains the following underlying data:
•
Catharanthus_roseus_v2.1_UT.cds (Predicted CDS). •
Catharanthus_roseus_v2.1_UT.gff (Genome annotation file (GFF)). •
Catharanthus_roseus_v2.1_UT.pep (Predicted proteins). •
Catharanthus_roseus_v2.1_UT.tr (Predicted transcripts). • Cuello et al – F1000R – SuppMat.xlsx (Supplementary tables). Data are available under the terms of the Creative Commons Attribution 4.0 International license (CC-BY 4.0).
