# Supplementary material for: An updated version of the Madagascar periwinkle genome
Source: F1000Res. 2022 Dec 21;11:1541. [Version 1] doi: 10.12688/f1000research.129212.1 (PMC9902796; doi:10.12688/f1000research.129212.1)
Supplement: Peer review history

# Peer review: Reviewer response for version 1

**DOI:** 10.5256/f1000research.141880.r158636

**Published:** 2023-02-06

**Authors:** Amit Rai

**Affiliations:** 1 Graduate School of Pharmaceutical Sciences, Chiba University, Chiba, Japan

In the presented article, authors reported an updated version of genome assembly for Madagascar periwinkle, which is a valuable model plant species to study MIA biosynthesis. Compared to the previously published genome assemblies for Madagascar periwinkle, this study used long read sequencing technology and achieved an improvement in terms of contig N50.

 Without a doubt, this is a better genome assembly, but authors should have considered scaffolding through HiC to achieve a chromosome-scale genome assembly as that would have allowed them to discover novel features contributing MIA biosynthesis and evolution.

 Nevertheless, the resource presented here is valuable, and will inspire researchers to combine the generated datasets in this study with new sequencing data to derive a chromosome-scale genome assembly for *C. roseus*. For these reasons, I support its indexing.

Are the datasets clearly presented in a usable and accessible format, and the assembly and annotation available in an appropriate subject-specific repository?

Yes

Are sufficient details of the sequencing and extraction, software used, and materials provided to allow replication by others?

Yes

Are the rationale for sequencing the genome and the species significance clearly described?

Yes

Are the protocols appropriate and is the work technically sound?

Yes

Reviewer Expertise:

Genome sciences, Metabolomics research. Evolutionary biology, MIA biosynthesis pathways

I confirm that I have read this submission and believe that I have an appropriate level of expertise to confirm that it is of an acceptable scientific standard.

# Peer review: Reviewer response for version 1

**DOI:** 10.5256/f1000research.141880.r158640

**Published:** 2023-01-04

**Authors:** Evangelos Tatsis

**Affiliations:** 1 Chinese Academy of Sciences Centre for Excellence in Molecular Plant Sciences, Shanghai Institute of Plant Physiology and Ecology, Shanghai, China

The current work reports an updated version of the significant medicinal plant *Catharanthus roseus*. *C. roseus* is the only source of the clinically used anticancer drug vinblastine and such work can provide resources for molecular breeding to improve the production of vinblastine and other MIAs.

 The sequencing techniques and the bioinformatic analysis are appropriate. I recommend the current manuscript for indexing as it is.

Are the datasets clearly presented in a usable and accessible format, and the assembly and annotation available in an appropriate subject-specific repository?

Yes

Are sufficient details of the sequencing and extraction, software used, and materials provided to allow replication by others?

Yes

Are the rationale for sequencing the genome and the species significance clearly described?

Yes

Are the protocols appropriate and is the work technically sound?

Yes

Reviewer Expertise:

Plant specialised metabolism

I confirm that I have read this submission and believe that I have an appropriate level of expertise to confirm that it is of an acceptable scientific standard.
